# Dental fear and anxiety in Estonian and Vietnamese schoolchildren: A comparative study of two populations

**DOI:** 10.1002/cre2.127

**Published:** 2018-08-07

**Authors:** Minh Son Nguyen, Thuy Trang Nguyen, Bui Bao Tien Nguyen, Mare Saag, Jana Olak

**Affiliations:** ^1^ Danang University of Medical Technology and Pharmacy Vietnam; ^2^ Institute of Dentistry University of Tartu Estonia

**Keywords:** dental anxiety, dental caries, dental fear, schoolchildren

## Abstract

Dental fear and anxiety (DFA) has the impact on the development of dental caries. Ethnic background and oral health care system may contribute to DFA model. The aim of the study was to compare DFA in relation to dental health between Estonian and Vietnamese schoolchildren and to determine DFA cutoff point for schoolchildren of two countries. The sample comprised 900 schoolchildren (344 from Estonia and 556 from Vietnam). DFA was measured by using 11 fear items of the modified Dental Subscale of the Children's Fear Survey Schedule (CFSS‐DS). Dental health was recorded by using the dental caries experience index of mixed dentition (dmft/DMFT). Results showed that the mean score of dmft/DMFT in Estonian and Vietnamese schoolchildren was 5.2 ± 3.1 and 4.1 ± 3.2, respectively. The mean score of 11‐item CFSS‐DS of Vietnamese schoolchildren (20.8 ± 9.1) was significantly higher compared with Estonian schoolchildren (15.4 ± 4.4, *p* < 0.001). The DFA cutoff point of 11‐item CFSS‐DS in Estonian schoolchildren was 17.5, whereas in Vietnamese, it was 24.5. The lower ranking of DFA was significantly associated with Estonian schoolchildren who had more FT (*p* < 0.001). In conclusion, the level of DFA of schoolchildren was different in Estonia and Vietnam. Vietnamese schoolchildren had higher DFA scores and cutoff point of the modified CFSS‐DS than Estonian schoolchildren. The obtained results suggest that DFA in schoolchildren can be related to oral health care system of each country.

## 1 INTRODUCTION

Dental fear is defined as a specific anxiety referring to threatening stimuli in the dental situation, and anxiety is a feeling of fear (Armfield, Stewart, & Spencer, 2007). The concepts of dental fear and dental anxiety have often been used interchangeably in the dental literature. The term “dental fear and anxiety” (DFA) is used throughout this study to address the fear of visiting a dentist for receiving dental treatment.

Prevalence of DFA among children varies in different areas. It is estimated that 20–50% of Asian children have DFA (Lee, Chang, & Huang, 2007; Paryab & Hosseinbor, 2013; Raj, Agarwal, Aradhya, Konde, & Nagakishore, 2013), whereas 6–20% European children self‐reported fear of dental treatment (Klingberg & Broberg, 2007; Luoto, Lahti, Nevanperä, Tolvanen, & Locker, 2009; Olak et al., 2013). Hence, the oral health care system could have some significance in the management of DFA among children. It has been known that DFA is a factor contributing to high risks of dental caries and poor oral health among children (Eitner, Wichmann, Paulsen, & Holst, 2006; Luoto et al., 2009; Olak et al., 2013). The findings of previous studies have demonstrated that DFA has a close connection with the frequency of visiting a dentist, especially if the child received local anesthesia (i.e., pulpectomy, restorations, or extractions). Fearful children often avoid or delay the appointment of dental treatment, which may increase the existing serious oral problems (Armfield et al., 2007; Luoto et al., 2009).

The consequence of DFA has an adverse impact on quality of life, psychological function, and social communication. Determining the threshold of fear and anxiety is significant in dental patient management. The Dental Subscale of the Children's Fear Survey Schedule (CFSS‐DS) is commonly applied in DFA studies (Gustafsson, Arnrup, Broberg, Bodin, & Berggren, 2010; Lee, Chang, & Huang, 2008; Nakai et al., 2005; ten Berge, Hoogstraten, Veerkamp, & Prins, 1998). The original version of CFSS‐DS has 15 questions that show reliable and valid responses in evaluating DFA (Cuthbert & Melamed, 1982). However, such items as fear of a “doctor,” “having a stranger touch you,” “having to go to the hospital,” “people in uniform,” and “having somebody looking at you” were ambiguous for DFA. Therefore, Rantavuori, Lahti, Seppä, and Hausen (2005) modified the original CFSS‐DS into an 11‐item questionnaire for more focus on dentistry. The modified CFSS‐DS has been validated and used in previous studies (Luoto et al., 2009; Olak et al., 2013; Rantavuori, Tolvanen, & Lahti, 2012).

DFA is an unpleasant emotion from psychological impacts. Previous studies demonstrated that DFA was different by age and gender (Lee et al., 2007; Paryab & Hosseinbor, 2013). This tends to suggest that there will be a stronger focus on biological factors as related to the contribution of DFA. The findings in various ethnic groups revealing different genetic factors, physical growth, and sociocultural features in the seeking of dental treatment can support the DFA model. In addition, the economic differences between high‐income and lower‐middle‐income countries may relate to a policy of child welfare and health care; all of which will influence the oral health care program for the children. Hence, there is a need for systematic collection and comparison of data on DFA from populations in different countries.

It is unknown whether DFA in Asian children differs from that in European children. Given the importance of DFA among children, the present study implemented data collection of the dental status and DFA among schoolchildren who visited dentists in Estonia and Vietnam. Our hypothesis was that differences between ethnic groups and the oral health care systems might have an impact on DFA in children. Therefore, the aims of this study were (a) to compare DFA in relation to dental health between Estonian and Vietnamese schoolchildren and (b) to determine the cutoff point DFA of the 11‐item CFSS‐DS of the two countries.

## 2 METHODS

### 2.1 Setting

Estonia is a high‐income country located in Northern Europe, with approximately 1.3 million of the population in 2010. During the period of 2007–2013, the health care expenditure was 5.7% of gross domestic product (GDP per capita in 2013 = $19,072) and expenses for dental treatment form 3.5% of total healthcare costs (World Bank, 2018). The density of physicians and dentists (per 10,000 population) in Estonia was 32.4 and 8.9, respectively (World Health Organization, 2015). The Estonia insurance policy will cover for all dental treatment procedures in children up to 19 years and professional preventive checkup of oral health in 6‐, 9‐, and 12‐year‐old children (Kahur et al., 2008).

Vietnam is categorized in the lower‐middle‐income country located in Southeast Asia, with an estimated 94.6 million inhabitants (in 2010). The rate of growth in national health expenditures was accounted to be 6.0% of GDP; however, GDP per capita in Vietnam is only $1,871 as reported in 2013 (World Bank, 2018). The density of physicians and dentists (per 10,000 population) in Vietnam was 11.9 and 0.74, respectively (Nguyen, Witter, Bronkhorst, Truong, & Creugers, 2010; World Health Organization, 2015). Schoolchildren will receive 80% coverage of expenses for medical examination and treatment from Vietnam Social Security, but the coverage for high‐technique treatment in dentistry such as prosthodontic and orthodontic is excluded from Vietnam Social Security (Tien, Phuong, Mathauer, & Phuong, 2011).

### 2.2 Study sample size

Eight to 10‐year‐old schoolchildren with mixed dentition were selected for the current study because they were likely to experience dental treatment of decayed deciduous and first permanent molar teeth, meaning that they are fully conscious to answer questions related to DFA.

The schoolchildren were selected from southeastern Estonia because the dental caries experience index of children in this region is one of the highest in this country (Olak, Mändar, Karjalainen, Söderling, & Saag, 2007). Five hundred twenty‐two schoolchildren (*n* = 522) of 10 primary schools from Elva, Lähte, Melliste, Nõo, Räpina, Rõngu, Tartu, Tõrva, Võru, and Võnnu cities were invited to participate the study. Of the total sample, 485 schoolchildren participated dental examination and 344 replied the DFA questionnaire. The final Estonian sample recruited in the current study was 344 schoolchildren (response rate = 65.9%). All participants in each school were invited to the Tartu University Hospital for the dental examination. As the data collection of each group had to be carried out in short time, the accompanying teachers were invited to support the research team to interview schoolchildren about DFA while they were waiting for dental examination. All teachers were trained to use DFA questionnaire before the study was performed.

Danang city, located in central Vietnam, was selected for the data collection for the reason that the Danang's population is equivalent with Estonian. In addition, Danang is one of the few cities in Vietnam that implements the School Oral Health Promotion Programme (Nguyen, Nguyen, Nguyen, Olak, & Saag, 2016); therefore, schoolchildren had dental treatment experience. Six hundred schoolchildren from five primary schools were recruited for examining the dental status and interviewing DFA. Of the Vietnamese sample, 556 schoolchildren agreed to participate in our study (response rate = 92.6%). Each school in Vietnam has a dental office. During the school days, dentists would alternatively invite schoolchildren for dental examination and DFA interview. Table 1 shows the distribution of the respondents by age and gender of the two countries.

**Table 1:** Distribution of schoolchildren according to age and gender (*N* = 900)

	Estonia		Vietnam	
	*n*	%	*n*	%
Years of age				
8	110	32.0	223	37.0
9	174	50.6	146	35.6
10	60	17.4	187	27.4
Gender				
Boy	188	54.7	239	43.0
Girl	156	45.3	317	57.0
Total	344	100	556	100

### 2.3 Measuring DFA

Children's DFA was evaluated by using a modified CFSS‐DS questionnaire (Rantavuori et al., 2005). The most important modification was that the following eight items of fear were taken from 15 items of the original CFSS‐DS: “fear of keeping the mouth open,” “fear of the dentist,” “fear of teeth being cleaned by a professional,” “fear of hearing the sound of drilling,” “fear of not being able to breathe,” “fear of the dentist drilling,” “fear of dental injection,” and “fear of instruments put in the mouth”; also, three items were added: “fear of pain during dental treatment,” “fear of suction using the mouth,” and “dental fear in general.” The first 10 out of 11 items of the modified CFSS‐DS were divided into two groups: the first five items represented noninvasive or less invasive fear, and the remaining five questions focused on more invasive fear (Table 2).

**Table 2:** Comparison of dental caries experience among Estonian and Vietnamese schoolchildren (*N* = 900)

Dental caries experience	Estonia (*n* = 344)	Vietnam (*n* = 556)	*p*
Number of schoolchildren (%) with dental caries experience^a^			
dt/DT > 0	183 (53.2)	468 (84.2)	<0.001*
mt/MT > 0	10 (2.9)	4 (0.7)	0.021*
ft/FT > 0	303 (88.1)	155 (27.9)	<0.001*
dmft/DMFT > 0	320 (93.0)	489 (87.9)	0.026*
Mean number of teeth (*SD*) with dental caries experience^b^			
dt/DT	1.3 (1.8)	3.6 (3.0)	<0.001*
mt/MT	0.03 (0.2)	0.01 (0.1)	0.032*
ft/FT	3.8 (2.6)	0.4 (0.9)	<0.001*
dmft/DMFT	5.2 (3.1)	4.1 (3.2)	<0.001*

a Chi‐square test.

b Student's *t* test.

* Significant.

The five response categories of the Likert scale related to the increasing level of fear (score 1 = *not afraid at all* and score 5 = *very afraid*) were used for each item of the modified CFSS‐DS. In case a child had not experienced an item, this item was scored 0 and eliminated from statistical analysis. Thus, the total fear score of the 11‐item CFSS‐DS ranged from 11 to 55.

The answers to the question “dental fear in general” were dichotomized into “no fear” (Likert score ≤ 2) and “fear” (Likert score ≥ 3) category. The cutoff point of DFA was based on the receiver operating characteristic (ROC) analysis of item “dental fear in general” and the total score of the 11‐item CFSS‐DS. The optimal cutoff point on the ROC curve is determined by the maximum value of the sum of sensitivity and specificity.

The modified CFSS‐DS was translated from English into the native languages (Estonian and Vietnamese) and back‐translations were done to ensure agreement with the original form. Both versions were pretested for clarity among a group of schoolchildren. Children were asked about each item of 11‐item CFSS‐DS by the accompanying teachers (in Estonia) or the dentists (in Vietnam).

### 2.4 Recording dental health

The component of decayed teeth (dt/DT), missing teeth (mt/MT), and filled teeth (ft/FT) in mixed dentitions was recorded. The indices were then combined to describe dental caries experience and dental health of mixed dentition (dmft/DMFT). Each index of dental caries was classified into two groups (i.e., dt/DT > 0 and dt/DT = 0) to represent the dental treatment experience among schoolchildren. These indices were then analyzed to compare dental health and reflect association with DFA between two countries.

Dental examinations were carried out in the dental offices of each school. In Estonia, schoolchildren's dental health was recorded by four calibrated examiners, and the Kappa values of the consistency between interexaminer and intraexaminer were above 0.9. In Vietnam, the intraexaminer and interexaminer calibrations were performed by two dentists, and the calculated Kappa values were above 0.85.

The teachers and the parents of the children of the selected schools in both countries were informed of the survey. Written informed consent was obtained from all parents. This cross‐sectional study is a research cooperation program between the University of Tartu in Estonia and Danang University of Medical Technology and Pharmacy in Vietnam. The first procedure of collecting data started in Estonia (2008) and then continued in Vietnam (2014). The Ethics Committee of the University of Tartu (No. 166/T‐7 2008) and the Human Research Ethics Committee of Danang University of Medical Technology & Pharmacy (No. 523/CN‐DHKTYDDN 2014) approved the study. All procedures were performed according to the World Medical Association Declaration of Helsinki.

### 2.5 Statistical analysis

Data entry and statistical analysis were performed by version 17.0 of the Statistical Package for Social Sciences (SPSS). The Chi‐square test and Student's *t* test were to determine the differences in dental health and DFA between Estonian and Vietnamese schoolchildren. The Mann–Whitney *U* test was used to analyze the ranking of dental fear in two countries with regard to dental caries components. Cronbach's *α* and the ROC curve were used to assess the reliability of items and to determine the optimal cutoff point of the 11‐item CFSS‐DS. The confidence level at 95% and a two‐sided *p* value of 0.05 were used for a significant difference.

## 3 RESULTS

Among 900 schoolchildren aged 8–10 years (344 in Estonia and 556 in Vietnam), the prevalence of Estonian and Vietnamese schoolchildren with dental caries experience was 93.0% and 87.9%, respectively. The mean score of dmft/DMFT was higher in Estonian (5.2 ± 3.1) than in Vietnamese schoolchildren (4.1 ± 3.2, *p* < 0.001). FT was significantly dominant among Estonian schoolchildren (ft/FT > 0 = 88.1%, mean 3.8 ± 2.6, *p <* 0.001). Decayed teeth in Vietnamese schoolchildren (dt/DT > 0 = 84.2%, mean 3.6 ± 3.0) were significantly more common than in Estonian schoolchildren (53.2%, mean 1.3 ± 1.8, *p <* 0.001; Table 2).

The Likert scale ≥3 was used to determine fear among schoolchildren in each item. Children's general fear of dentistry amounted to 6.1% in Estonian and 14.7% in Vietnamese schoolchildren. The prevalence of other fear items was significantly higher in Vietnamese than in Estonian schoolchildren (*p* < 0.05), except for dental injection fear (*p* > 0.05). The mean score of noninvasive, invasive, and 11‐fear items of Estonian schoolchildren was 5.7 ± 1.5, 8.2 ± 3.3, and 15.4 ± 4.4, respectively, which was lower compared with Vietnamese schoolchildren (8.4 ± 3.6, 10.7 ± 5.1, and 20.8 ± 9.1, respectively, *p* < 0.05). “Fear of dental injections,” “fear of dentist drilling,” and “pain during dental treatment” were the most fearful items of the modified CFSS‐DS reported by schoolchildren of each country (the scores ranging from 2.0 to 2.7; Table 3).

**Table 3:** Comparison of percentage of schoolchildren with the Likert scale ≥3 and the mean score of fear items between Estonia and Vietnam (*N* = 900)

Fear of	Percentage of schoolchildren with the Likert scale ≥ 3^a^		*p* b	Mean (*SD*) of fear items		*p* c
	Estonia	Vietnam		Estonia	Vietnam	
Dental fear in general	6.1	14.7	<0.001*	1.5 (0.7)	1.9 (1.1)	<0.001*
All noninvasion items				5.7 (1.5)	8.4 (3.6)	<0.001*
Keeping the mouth open	0.6	8.1	<0.001*	1.1 (0.4)	1.4 (0.9)	<0.001*
Dentist	2.9	7.5	0.004*	1.2 (0.6)	1.4 (0.8)	0.002*
Teeth being cleaned by a professional	3.3	7.0	0.011*	1.2 (0.6)	1.3 (0.8)	0.057
Hearing the sound of drilling	3.0	21.3	<0.001*	1.2 (0.6)	1.9 (1.3)	<0.001*
Not being able to breathe	4.5	42.2	<0.001*	1.2 (0.7)	2.6 (1.7)	<0.001*
All invasion items				8.2 (3.3)	10.7 (5.1)	<0.001*
Instruments put in the mouth	3.2	19.3	<0.001*	1.3 (0.6)	1.9 (1.2)	<0.001*
Suction using the mouth	3.1	13.6	<0.001*	1.2 (0.6)	1.6 (1.1)	<0.001*
Pain during dental treatment	21.1	29.2	0.011*	2.0 (1.0)	2.3 (1.4)	<0.001*
Dentist drilling	26.4	33.2	0.036*	2.1 (1.1)	2.4 (1.5)	<0.001*
Dental injections	38.1	43.0	0.185	2.5 (1.3)	2.7 (1.5)	0.013*
Eleven fear items				15.4 (4.4)	20.8 (9.1)	<0.001*

a Children without experience of each fear item were excluded from the statistics.

b Chi‐square test.

c Student's *t* test.

* Significant.

The mean rank of all fear items with dt/DT, ft/FT, and dmft/DMFT among Estonian schoolchildren was significantly lower than among Vietnamese schoolchildren (*p* < 0.001; Table 4). Cronbach's *α* value in Table 5 showed the reliability of children's answers in both countries. The DFA optimal cutoff point of 11‐item CFSS‐DS of Estonian schoolchildren was 17.5 with 73% of sensitivity and 100% of specificity, whereas in the case of Vietnamese schoolchildren, the respective values were 24.5, 81.5%, and 86.5%, respectively. Regarding the optimal cutoff point, the prevalence of DFA in Estonian schoolchildren (30.7%) was statistical equivalence compared with Vietnamese schoolchildren (28.0%, *p* > 0.05).

**Table 4:** Comparison of the mean rank of the 11‐item CFSS‐DS according to dental caries experience between Estonian and Vietnamese schoolchildren (*N* = 900)

Dental caries experience	Number of cases	Mean rank		*p* a
		Estonia	Vietnam	
dt/DT > 0	419	169.2	225.6	<0.001*
mt/MT > 0	12	5.8	7.9	0.368
ft/FT > 0	307	139.2	180.4	<0.001*
dmft/DMFT > 0	523	218.0	289.9	<0.001*

a Mann–Witney *U* test.

* Significant.

**Table 5:** DFA cutoff point of the 11‐item CFSS‐DS in Estonian and Vietnamese schoolchildren

Variable	Estonia	Vietnam	*p*
Cutoff point	17.5	24.5	
Sensitive	73%	81.5%	
Specificity	100%	86.5%	
Cronbach's α	0.75	0.84	
Area under the curve	0.92	0.91	<0.001*
Number (%) schoolchildren with DFA^a^	66 (30.7)	104 (28.0)	0.512

*Note*. DFA: dental fear and anxiety.

a Number of children with the score CFSS‐DS above the cutoff point; Chi‐square test.

* Significant.

## 4 DISCUSSION

Dental fear is considered to be the predictor of high‐risk caries (Rantavuori, Lahti, Hausen, Seppä, & Kärkkäinen, 2004). The negative previous dental treatment often precedes DFA among children. Anxious children are likely to postpone or avoid a dental checkup; because of the “vicious circle of avoidance,” children are faced with more serious dental problems and more anxiety when they approach dental treatment (Armfield et al., 2007). Although the schoolchildren represented narrowly defined regions in each of the countries, the present study shows that DFA among Estonian schoolchildren differs from Vietnamese schoolchildren.

At 8–10 years of age, children are in a mixed dentition stage, which is a risk of caries lesions; therefore, they could have experienced dental treatment. Regarding the dental caries experience among Estonian and Vietnamese schoolchildren, these two populations concurred on a moderate level of dmft/DMFT, but the score of dmft/DMFT was higher in Estonian schoolchildren than Vietnamese schoolchildren. This can be interpreted that the Estonian schoolchildren sample was chosen from the regions having the highest dental caries index in Estonia (Olak et al., 2007), and the Vietnamese schoolchildren were from the city that the School Oral Health Promotion Programme with dental education and fluoride rinsing is being implemented (Nguyen et al., 2016).

The dmft/DMFT index reflects not only dental caries of the children but also the awareness of children's dental care by parents and the quality of oral health care system in a country. The fact that Vietnamese schoolchildren presented more untreated decayed teeth, on the other hand, the Estonian group had more filled teeth. This interesting finding can clarify some factors related to sociodemographic characteristics and oral health care systems contributing to DFA in children.

The difference of dt/DT and ft/FT between Estonian and Vietnamese schoolchildren might be explained by differences in the health care systems. Estonia belongs to the European Union where dental treatment is the least supported by the State, and the Estonian Health Insurance Fund covers all the dental treatment procedures in children up to 19 years, and professional preventive checkups of oral health at 6, 9, and 12 years of age is mandatory in Estonia (Kahur et al., 2008). Social health insurance in Vietnam supports medical care for children under 6 years of age, and until now, it has not focused on specialized dental care services (Tien et al., 2011).

Comparison of the DFA level between Vietnamese and Estonian schoolchildren revealed that more decayed teeth presented in dentition result in higher DFA in schoolchildren. Children with a history of pulpitis pain often experience dental anxiety and have behavioral problems during the treatment procedure (Jälevik & Klingberg, 2002). Another fact is that the parental attitude towards dental health care influences oral health behavior and dental perception in children. The parents of Vietnamese schoolchildren might often have the perception that primary teeth can be replaced by permanent teeth. For this reason, they tend to ignore taking their children to a dentist until children have an acute toothache due to a decayed tooth (Nguyen et al., 2016). In addition, the optional treatments for severely decayed primary teeth in Vietnam include medicine (i.e., NSAIDs or antibiotics) or extraction (Jacobsson, Ho Thi, Hoang Ngoc, & Hugoson, 2015); therefore, the experience of a persistent toothache and pain from extraction would increase DFA among children.

Our findings indicated that schoolchildren having more filled teeth had less dental fear. It is possible that these children may have been more familiar with the dental clinic environment; therefore, they were less afraid of the treatment procedures. The proof is that Estonian schoolchildren had more filled teeth and fewer untreated decayed teeth than their Vietnamese peers. It appears that the density of dentistry personnel per 10,000 of the Estonian population is 12 times higher than in Vietnam (Nguyen et al., 2010; World Health Organization, 2015). Therefore, Estonian parents might receive more dental instructions to increase awareness regarding the importance of the prevention of dental caries in their children and saving primary dentition, whereas in Vietnam, up to 60% of children never visit a dentist, and no more than 14% of children received a filling before 5 years of age (Jacobsson et al., 2015).

The findings of the present study are in line with other studies in that dental treatment with less invasive pain could create confidence intervals among children when visiting a dentist; in other words, low‐fear children were found to have experienced more checkup visits (Lee et al., 2008; Nakai et al., 2005; ten Berge, Veerkamp, & Hoogstraten, 2002).

The invasive dental techniques would cause DFA in children. The evidence that both Estonian and Vietnamese children self‐reported that the highest score items were drilling and dental injection, and these findings are consistent with other studies (El‐Housseiny, Alamoudi, Farsi, & El Derwi, 2014; Nakai et al., 2005; Raj et al., 2013; Rantavuori et al., 2012). The drilling of teeth and dental injections are often used in the dental treatment of caries, pulpitis, abscess, or extraction. Most children might experience severe procedure‐related pain in hospitals by needle injections; therefore, one has to consider negative emotional and psychological implications when having dental treatment. Numerous studies have indicated that noninvasive treatments (i.e., dental cleanings and atraumatic restorative treatment) reduce fear in children during the treatment procedure (Brukiene, Aleksejuniene, & Balciuniene, 2006; Leal, Abreu, & Frencken, 2009; Olak et al., 2013).

The scores in items of the modified CFSS‐DS were often higher among Vietnamese schoolchildren than among Estonian children. This suggests that different ethnic groups are significantly associated with DFA, and the DFA model depends on sociocultural differences and genetic factors. For instance, in the same age group, Finnish children had a higher score of each item in the 11‐item CFSS‐DS than Estonian children but lower than Vietnamese children (Rantavuori et al., 2012). In addition, fear and anxiety are the self‐protection of the body to avoid pain, and susceptible pain could be lower in Vietnamese than Estonian schoolchildren due to differences in physical growth. Wu and Hirsch (2010) demonstrated that Asian children had more frequently orofacial pain compared with European children.

The fact that we identified differences in the DFA threshold between the two groups is a key outcome of this study. Interestingly, Vietnamese schoolchildren had a higher DFA threshold than Estonian schoolchildren. The optimal cutoff point of DFA of Vietnamese schoolchildren was 24.5, whereas Estonian was only 17.5. The differences can be explained by some reasons. First, the DFA cutoff point is positively dependent on the mean total score of 11‐item CFSS‐DS. Second, the educational environment is likely to shape personality traits associated with the perception of fear in children. For instance, Taiwanese children had a cutoff score of the original CFSS‐DS higher compared with Swedish children at the same age of 8 years old (Gustafsson et al., 2010; Lee et al., 2007).

In the present study, the optimal cutoff scores of the modified CFSS‐DS were at high sensitive (73^_^81.5%) and high specificity (86.5–100%) for both Estonian and Vietnamese samples. These findings were in line with the study conducted in Taiwan (Lee et al., 2007) and Sweden (Gustafsson et al., 2010) that also demonstrated the reliability of the original CFSS‐DS instruments. The high sensitivity and specificity of 11‐item CFSS‐DS mean that cutoff point might apply in dental practice to identify a potential fear and anxiety of a child before dental treatment. In other words, children having DFA score above the cutoff point of 11‐item CFSS‐DS often exhibit anxiety or fear while receiving dental treatment. Therefore, dentists should have suitable counseling approach to reduce DFA in children.

When each DFA cutoff point was applied for schoolchildren in each country, the prevalence of high DFA among schoolchildren in Estonia (30.7%) and Vietnam (28.0%) is on the same level. It was comparatively high compared with the report of Lee et al. (2007) in Taiwan (20.6%); Chellappah, Vignehsa, Milgrom, and Lam (1990) in Singapore (17.7%); and Luoto et al. (2009) in Finland (11%). This is a challenge for both the Estonian and Vietnamese oral health systems because DFA is predictive of dental caries.

The use of the modified CFSS‐DS adapted for a particular language area also enhances assessment reliability. Our study confirmed that the cutoff point of modified CFSS‐DS was unique for each country. The cutoff point of DFA reported in previous studies is varying; for example, the cutoff point of 15 items of the original CFSS‐DS tends to increase in older children; it varies in the range of 38–42 scores and differs by ethnic groups (Gustafsson et al., 2010; Klingberg & Broberg, 2007). Therefore, it is necessary to verify the participants' characteristics before applying the cutoff point of CFF‐DS for the evaluation of DFA.

Applying the modified CFSS‐DS instrument is meaningful in the prediction of dental fear among children and supports the dentist while choosing the methods of communication with children before and during dental treatment. In the present study, we used the survey questionnaire to identify DFA and did not observe schoolchildren's attitudes and behavior during the dental treatment procedure. This must be regarded as a shortcoming of the study; also, the study needs to be extended to different age groups.

## 5 CONCLUSIONS

The level of DFA in schoolchildren was different in Estonia and Vietnam. Vietnamese schoolchildren had higher DFA scores and the cutoff point of 11‐item CFSS‐DS than Estonian schoolchildren. The obtained results suggest that DFA in schoolchildren can be related to oral health care system of each country.

## CONFLICT OF INTEREST

The authors state no conflict of interest.
